# Population receptive field size does not correspond to spatial frequency processing in scene-selective cortex

**DOI:** 10.1162/IMAG.a.1111

**Published:** 2026-01-29

**Authors:** Charlotte A. Leferink, Claudia Damiano, Dirk B. Walther

**Affiliations:** Department of Psychological and Brain Sciences, Dartmouth College, Hanover, NH, United States; Department of Psychology, University of Toronto, Toronto, Canada

**Keywords:** high-level visual cortex, PPA, scene representation, spatial frequency decoding, population receptive fields

## Abstract

Humans are experts at scene perception, for example, for recognizing familiar places or navigating new spaces. Scene representations in the brain are often thought of as global or large-scale, suggesting that scenes are represented by coarse-grained or holistic features. Recent work has shown that high spatial frequency or fine-grained visual information is also represented within scene-selective areas, which challenges the idea of strictly low-frequency, gist-like representations in those regions. Here, we explore whether these contrasting views can be explained by the size of the population receptive fields (pRFs) within two scene-selective areas: the parahippocampal place area (PPA) and occipital place area (OPA). Our results show that both the PPA and the OPA contain voxels with a variety of receptive field sizes, which follow a gradient from large to small along the anterior-posterior axis. This organization would predict scene representations in anterior PPA/OPA to be dominated by low spatial frequencies and scene representations in posterior PPA/OPA by high spatial frequencies. We find the opposite pattern when decoding scene categories of spatial frequency-filtered images. Our results indicate that preferred scene feature representations are transformed along the visual hierarchy, adhering closely to the expected correspondence between spatial frequency preferences and pRF size in early visual areas, but demonstrably less so in high-level visual brain regions.

## 1 Introduction

The retinotopic organization of visual cortex is one of the most important guiding principles for understanding how the organization of hundreds of millions of neurons that comprise human visual cortex modulate visual perception. A well-accepted principle of retinotopic mapping across the human visual cortex is that large receptive fields are associated with low spatial frequency representations and small receptive fields with high spatial frequency representations (Aghajari et al., 2020; Altan et al., 2024; Henriksson et al., 2008; Rovamo et al., 1978; Sasaki et al., 2001; Shulman & Wilson, 1987; Singh et al., 2000).

For example, primary visual cortex contains small receptive fields near the occipital pole that are related to a high sensitivity for fine spatial details (Aghajari et al., 2020; Henriksson et al., 2008). At the other end of the spectrum, due to decreased sensitivity to high spatial frequencies in peripheral pRFs (Lieber et al., 2023), scene-specialized regions such as the parahippocampal cortex (PHC), which contain large and peripheral pRFs (Arcaro et al., 2009; Hasson et al., 2002), are thought to process mainly low-spatial frequency information. Given the expansiveness of scenes, and that the PHC processes global or gist information, this idea is certainly conceptually attractive.

Perception of scene gist is thought to rely predominantly on global visual features, which are conveyed by the low spatial frequency (low SF) information of a scene (Greene & Oliva, 2009; Oliva & Torralba, 2006; Schyns & Oliva, 1994). This view finds support in the peripheral and large receptive field bias in the parahippocampal place area (PPA) (Arcaro et al., 2009; Hasson et al., 2002; Levy et al., 2001).

Neuroimaging studies, however, show a preference of the PPA for high spatial frequencies (Rajimehr et al., 2011), as well as for visual features requiring fine spatial resolution (Choo et al., 2017; Nasr et al., 2014; Walther et al., 2011), and even for high spatial frequency-filtered scenes (Berman et al., 2017; Kauffmann et al., 2014). How can this be the case if large receptive fields only resolve coarse or low spatial frequency information? Either scene-selective cortex does not solely contain large receptive fields, or receptive field size in high-level visual cortex is not indicative of the level of detail that can be extracted from a scene. These findings directly contradict the expectation that scene-selective visual cortex solely processes global and coarse-grained scene features.

Here, we present new evidence to reconcile these contradictory accounts. We combine high-resolution population receptive field mapping of scene-selective areas OPA and PPA with a multi-voxel pattern analysis of the content of spatial frequency filtered scene images. We show that spatial frequency selectivity in these regions is not determined by receptive field size but rather by an anatomical subdivision along the AP axis in the case of the PPA.

## 2 Methods

### 2.1 Participants

We analyzed data from 181 participants (109 females, 72 males) from the Human Connectome Project (HCP) dataset (Benson et al., 2018; Van Essen et al., 2013) that had been scanned at both 7T and 3T. The data of 24 participants were excluded from the analysis because we were unable to functionally localize their regions of interest using the data included in the HCP data set. We here present data from the remaining 157 participants (94 females, 63 males, age 22–36). All participants provide written and informed consent prior to data collection. All data utilized in this study have been approved by the Ethics and Review Board at the University of Toronto. We used previously published data from various sources that received ethics approval from their respective institutions. The HCP data were utilized in accordance with the HCP data use guidelines.

### 2.2 Functional localization of regions of interest

The HCP 3T scans included a working memory task of visual stimuli from several categories (i.e., places, faces, tools, and body parts). We used these data to define linear contrasts, allowing us to functionally localize high-level visual regions of interest (ROIs). Whole-brain data were collected at a 2 mm isotropic resolution with a time to repetition (TR) of 0.7 s. The memory task 3T scans were motion-corrected to the middle volume of the 3rd (of 6) runs. The 3T scan also contained a T1-weighted anatomical scan with a 0.7 mm isotropic resolution, which we used to align the data across scans. We computed the alignment of these data with the 7T retinotopy data (see below) using the T1-weighted anatomical scan from the 3T scanning sessions as an intermediary.

We defined regressors for the occurrence of places, faces, and objects (tools, in this case) based on the log files provided in the HCP database. We performed a regression analysis using Afni’s 3dDeconvolve (Cox & Jesmanowic, 1999) and obtained linear contrasts between these regressors, which were corrected for multiple comparisons using False Discovery Rate. We defined ROIs based on spatially contiguous clusters of voxels with significant contrast (at least *q* < .001) as follows: The parahippocampal place area (PPA) and the occipital place area (OPA) using “places > faces and objects” (Dilks et al., 2013; Epstein & Kanwisher, 1998); the fusiform face area (FFA) using “faces > places and objects” (Kanwisher et al., 1997); and the lateral occipital complex (LOC) using “objects > faces and places”. Note that LOC is typically defined using a contrast of “objects > scrambled objects” (Malach et al., 1995), but scrambled images were not available in the HCP working memory dataset. Further analysis did not find a large difference between a probabilistically defined LOC and the “objects > faces and places” contrast. We compare the four ROIs in our evaluation of the distribution of pRF representations of the viewing plane. We observe similar distributions as was previously found by Silson et al. (2015) and Silson, Groen, et al. (2016). We continued to conduct our analyses on only the two ROIs that are scene-specific, namely OPA and PPA, out of the four ROIs we initially localized.

### 2.3 Population receptive field analysis

The population receptive field (pRF) mapping experiment included in the 7T dataset consisted of 6 runs. Each run featured stimuli designed to stimulate the high-level visual cortex (Benson et al., 2018). Whole-brain data were collected at a 1.6 mm isotropic resolution with a time to repetition (TR) of 1 s.

The population receptive fields of each voxel were computed using the non-linear regression-based Compressive Spatial Summation algorithm (Kay et al., 2013). We converted the data from HCP’s grayordinates into voxel space using the Neuropythy toolbox (Benson & Winawer, 2018) and masked the pRF data with the functionally defined regions of interest for each participant (see above for details of the functional localizers). Averaged pRF model fits from the HCP dataset were calculated using a threshold of R^2^ > 10%, reflecting the optimized cutoff threshold of signal-to-noise ratio relative to the HCP dataset, as reported in Benson et al.’s (2018) results.

To obtain visual field plots, we projected each voxel onto the viewing plane by modeling its receptive field as a two-dimensional isotropic Gaussian distribution. The location of the mean of the Gaussian function was obtained by the pRF analysis in polar coordinates, that is, polar angle and eccentricity. The standard deviation of the Gaussian function served as a measure of the size of the pRF (Silson et al., 2021; Winawer et al., 2010). The visual field plots of the individual voxels within an ROI were combined by computing the maximum at each location in the viewing plane. The pixel values were then averaged separately within the left and right half of the viewing plane to quantify the ipsilateral and contralateral representations of the viewing plane for a given ROI.

### 2.4 Modeling the anatomical organization of pRF properties

To test the relationship between receptive field size and eccentricity, we conducted a mixed-effects model of pRF size, with eccentricity as a fixed effect and participants as the random effect within each of our ROIs. We further explored the retinotopic organization of voxels within each ROI. To this end, we computed a mixed-effects model of pRF size, with the Cartesian coordinates of the voxels as fixed effects and participants as the random effect.

We also calculated viewing plane biases in the field of view relative to the viewing plane for each voxel within each hemisphere of the ROI using the Winawer et al. (2010) method. We rendered the pRF of each voxel onto the viewing plane as an isotropic two-dimensional Gaussian function at the location specified by values for the eccentricity and polar angle. The pRFs for all relevant voxels were combined using a pixel-wise maximum function. Contralateral hemifield bias and lower versus upper hemifield bias were computed using two-sample t-tests of the average pixel weight for each hemisphere, and for the top versus the bottom half of the viewing plane, respectively.

### 2.5 Spatial frequency-dependent decoding of scene categories

To assess whether the distribution of spatial frequency selectivity within visual cortex relates to the distribution of receptive field sizes, we reanalyzed the data from Berman et al. (2017). In that study, 14 participants saw grayscale images of beaches, highways, forests, and cities in a block-design fMRI experiment. Images were filtered for low spatial frequencies (< 0.75 cycles per degree; low SF), high spatial frequencies (>6 cycles per degree; high SF). All three versions of the images were jointly normalized for equal luminance and contrast, according to the recommendations by Perfetto et al. (2020).

The PPA and OPA of participants in the Berman et al. (2017) dataset were defined using separate functional localizer scans. Here, we reanalyze the data of the 14 participants for whom all four ROIs could be localized bilaterally. In addition, we projected an atlas-based map of V1 (Wang et al., 2015) from MNI space into individual subject space. Furthermore, we projected the group-level map of receptive field size computed from the HCP data from MNI space into individual subject space. We separated the voxels in each ROI into equal-sized halves (median split) according to (i) their location along the AP-axis, or (ii) the size of their population receptive fields.

FMRI data from the experiment were corrected for motion, converted into percent signal change, and spatially smoothed with 2 mm FWHM. Residuals from a GLM analysis with only nuisance regressors for subject motion as well as scanner drift were used as features for the subsequent multi-voxel pattern analysis (MVPA). In the MVPA, we decoded scene categories in a leave-one-run-out cross-validation using a linear support vector machine classifier, separately for the low SF and high SF scenes and separately for each participant. Group-level decoding accuracy was computed as the mean across participants. We used a repeated-measures analysis of variance (ANOVA) to assess the differences between low SF and high SF decoding accuracy across the separate segments (anterior vs. posterior, and small vs. large receptive fields), separately within the OPA, PPA, and within V1. Post hoc analyses were conducted using Tukey tests.

We also analyzed the fMRI data using univariate percent signal change analysis. We calculated the percent signal change with respect to the mean signal of each run for each participant. Through investigating both the univariate and the multivariate results, we can measure not only the level of mean neural activity but also the strength of the scene-category signal elicited by the image content contained in a particular spatial frequency band.

## 3 Results

Retinotopic fits for the 7 Tesla retinotopy Human Connectome Project dataset (Benson et al., 2018) were generated using the Compressive Spatial Summation algorithm (Kay et al., 2013). The analysis yielded estimates of polar angle, size, eccentricity, and voxel fit measures. We used a threshold of adjusted R^2^ > 0.1 across all ROIs in our hemifield analysis for the voxels within functionally defined regions of interest (ROIs): PPA, LOC, OPA, and FFA.

**Fig. 1.**
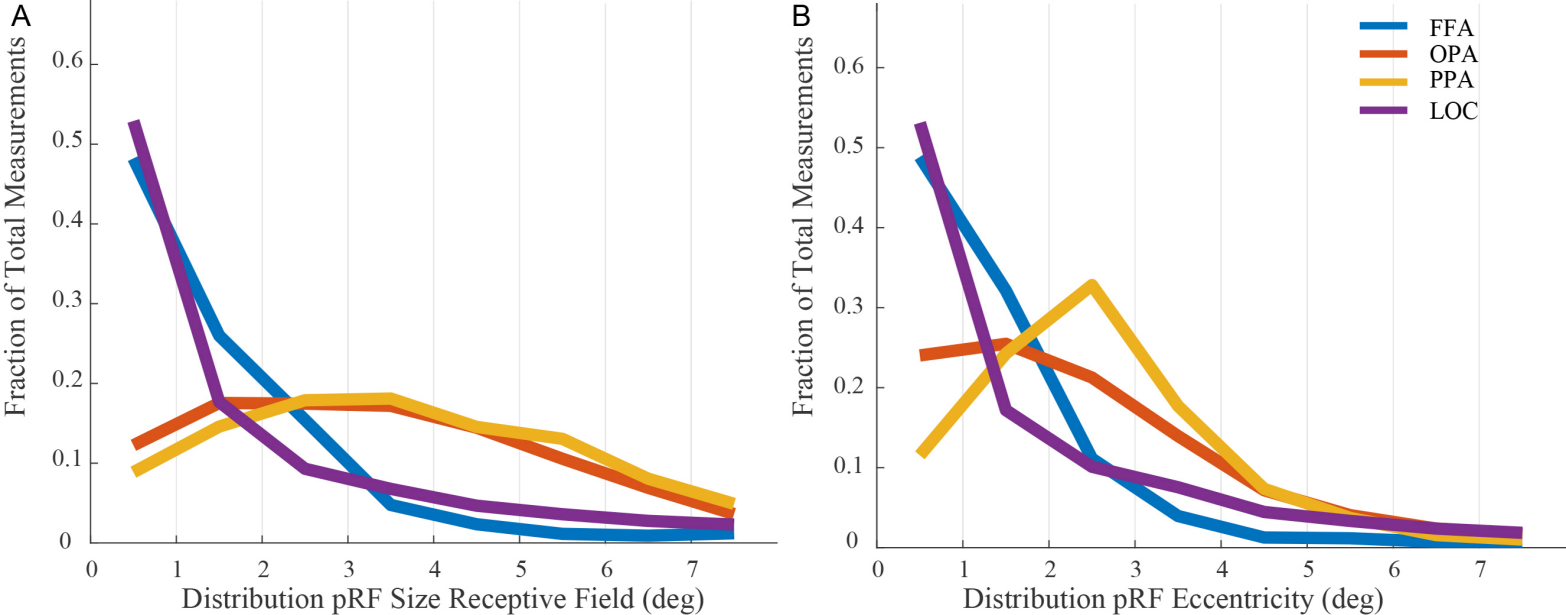
Distribution of pRF size (A) and eccentricity (B) for each of the four ROIs across bins of viewing angle. Each bin was normalized within each participant prior to averaging.

The normalized histograms of the distributions showed that the FFA and the LOC contain many voxels with small receptive fields and eccentricity near the fovea (Fig. 1). By comparison, the distributions of eccentricity and receptive field size were skewed toward larger receptive fields and higher eccentricity in the PPA and the OPA (Fig. 1). Nevertheless, 12.9% of the OPA voxels and 19.5% of the PPA voxels have pRFs with an eccentricity of less than 1 degree of visual angle, which is considered to be within the range of high-acuity foveal vision (Wandell, 1995).

Consistent with previous studies (Kay et al., 2013; Wandell & Winawer, 2015), we found that the relationship between eccentricity and size of pRFs was significantly positive in each of the four ROIs. A mixed-effects model of pRF size as a function of eccentricity, with participants as a random factor showed a linear relationship in each ROI in each hemisphere (all *p* < .001), see Supplementary Figure S1.

### 3.1 Projections onto the viewing plane

**Fig. 2.**
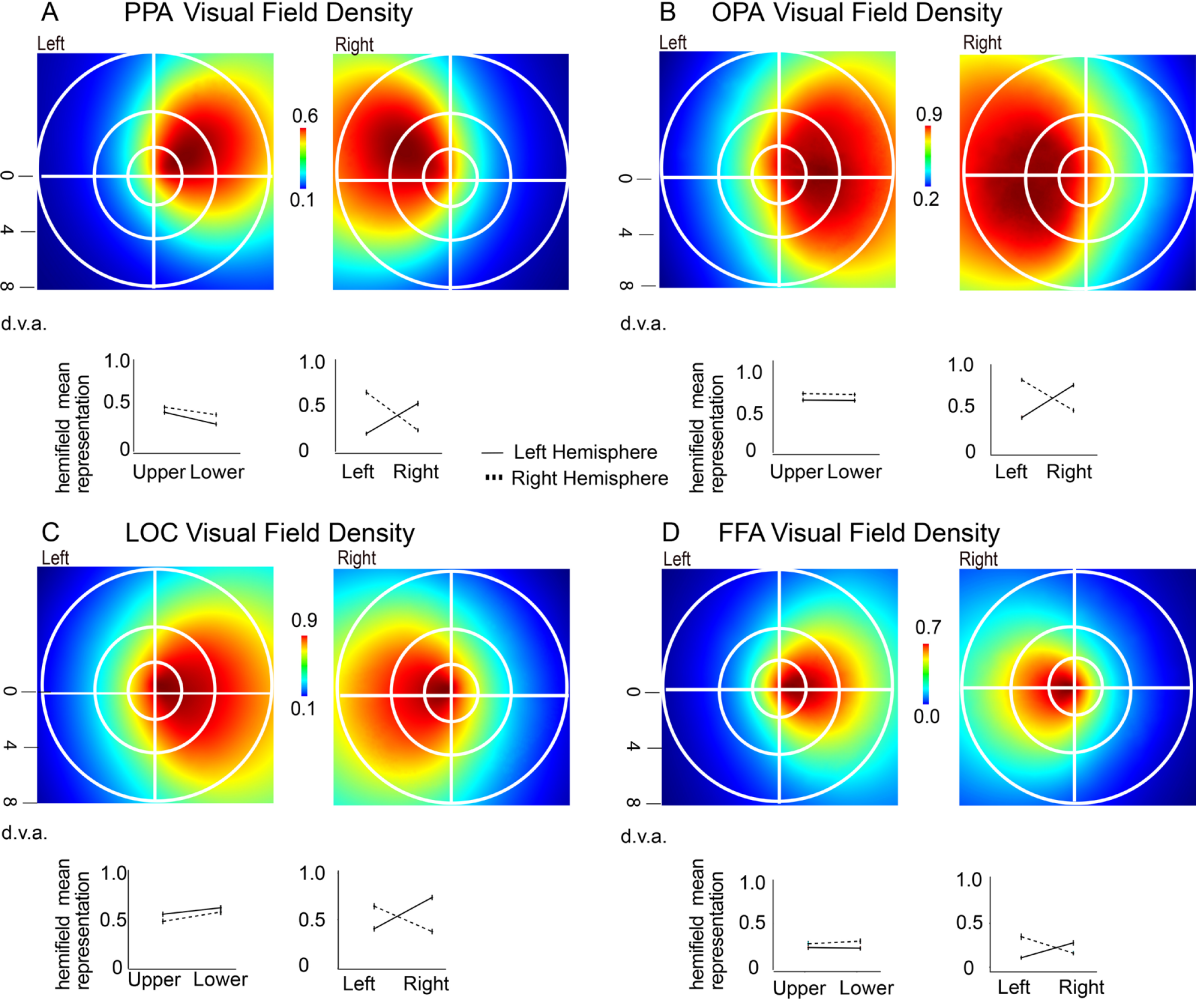
(A) PPA; (B) OPA; (C) LOC; (D) FFA. Topographical characterization showing distribution of pRF values across hemispheres (top), average pixel weight for each hemifield (middle), and anatomical organization of average size of receptive field (bottom) for each ROI.

We calculated the viewing plane bias using each of the pRF mappings for each of the four ROIs. We projected the pRF for each voxel of the left and right hemispheres onto the viewing plane, using a maximum operation to combine pRFs from multiple voxels (Winawer et al., 2010). We then computed a two-sample t-test to compare the values of the pixels in the left versus right hemifield as well as the upper versus lower hemifield. The PPA (right: *M* = 0.94 (0.22), left: *M* = -0.86 (0.28)), *t*(86) = -28.72, *p* < .001, and the FFA (right: *M* = 0.86 (0.33), left: *M* = -0.63 (0.56)), *t*(69) = -9.50, *p* < .001, showed significant contralateral bias in both hemispheres. Consistent with previous studies (Silson, Groen, et al., 2016; Silson et al., 2015), the LOC and OPA also had a significant contralateral bias (LOC – right: *M* = 0.8154 (0.23), left: *M* = -0.66 (0.34), *t*(121) = -21.13, *p* < .001; OPA – right: *M* = 0.86 (0.25), left: *M* = -0.80 (0.31), *t*(146) = -31.56, *p* < .001; see Fig. 2). We found an upper hemifield bias for the left PPA: *t*(86) = 8.44, *p* < .001 and the right PPA *t*(86) = 6.76, *p* < .001. There was no significant difference in degree of upper hemifield bias between the anterior segment and the posterior segment of PPA (Supplementary Fig. S2a, b). LOC showed a lower-field bias (Fig. 2), on the left: *t*(121) = -5.86, *p* < .001 and on the right: *t*(121) = -2.79, *p* < .001. No significant upper or lower hemifield bias was found within the left FFA, *t*(69) = 0.90, *p* = .37, but there was a significant lower hemifield bias in the right FFA, *t*(69) = -2.79, *p* = .0068. The OPA did not show a significant upper or lower hemifield bias in either hemisphere (left: *t*(150) = 0.37, *p* = .71 and right: *t*(150) = 1.09, *p* = .29). However, after splitting the OPA using a median split along the anterior-posterior axis, we found no upper or lower hemifield bias: *p* = .289; *t*(146) = 1.065, *M_diff_* = 0.035 (0.397) in the anterior OPA segment (Supplementary Fig. 2a). The posterior OPA segment (Supplementary Fig. 3b) shows a significant lower hemifield bias: *p* < .001, *t*(146) = -4.069, *M_diff_* = -0.141 (0.419), which is consistent with past results of a lower hemifield bias in OPA (Silson et al., 2015).

### 3.2 pRF properties along the anatomical axes

We explored the anatomical distribution of pRF properties by mapping them onto the three cardinal neural axes: medial to lateral, inferior to superior, and anterior to posterior. A mixed-effects model of the distribution of pRF eccentricity along the lateral-medial axis, with participants as a random effect, showed a more foveal representation laterally than medially, in PPA, OPA, and FFA, but not LOC, for which we found the reverse (Fig. 3b). Accordingly, pRF size also increased from lateral to medial in PPA and FFA and from medial to lateral in LOC. Both eccentricity and pRF size increased from inferior to superior in both the PPA and the OPA (Fig. 3a). Along the anterior to posterior axis, we observed a shift from smaller and more foveal representations posteriorly to larger and more peripheral representations anteriorly in both the PPA and the OPA. The change in pRF size along the A-P axis is more robust (larger t statistic) than the change in eccentricity (Fig. 3a and 3c; Supplementary Table S1). We also checked whether there was a reduced R^2^ moving from posterior to anterior. The average R^2^ values after thresholding at 10% variance explained (with standard deviation in brackets) for the PPA and OPA are the following: PPA anterior = 0.1538 (0.0678), PPA posterior = 0.1541 (0.0678), OPA anterior = 0.2491 (0.1130), and OPA posterior = 0.2494 (0.1132). Averaged pRF model fits from the HCP dataset were calculated using a threshold of R^2^ > 10% to produce an average sigma mask.

**Fig. 3a.**
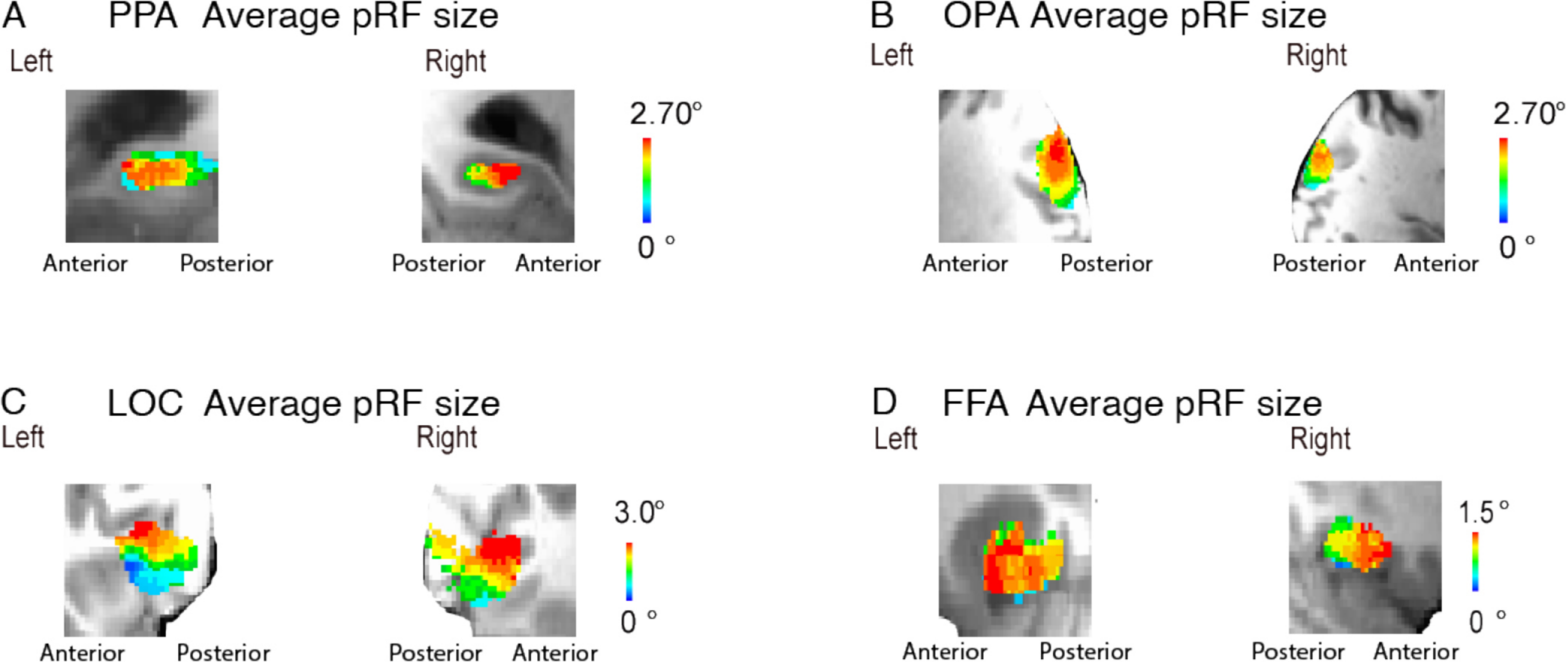
(A) PPA; (B) OPA; (C) LOC; (D) FFA. Organization of average size of receptive field (bottom) for each ROI. Direction and relative magnitude of gradient of increasing pRF size is indicated by arrow. Gradient of pRF size was significant across all ROIs, *p* < .001.

**Fig. 3b:**
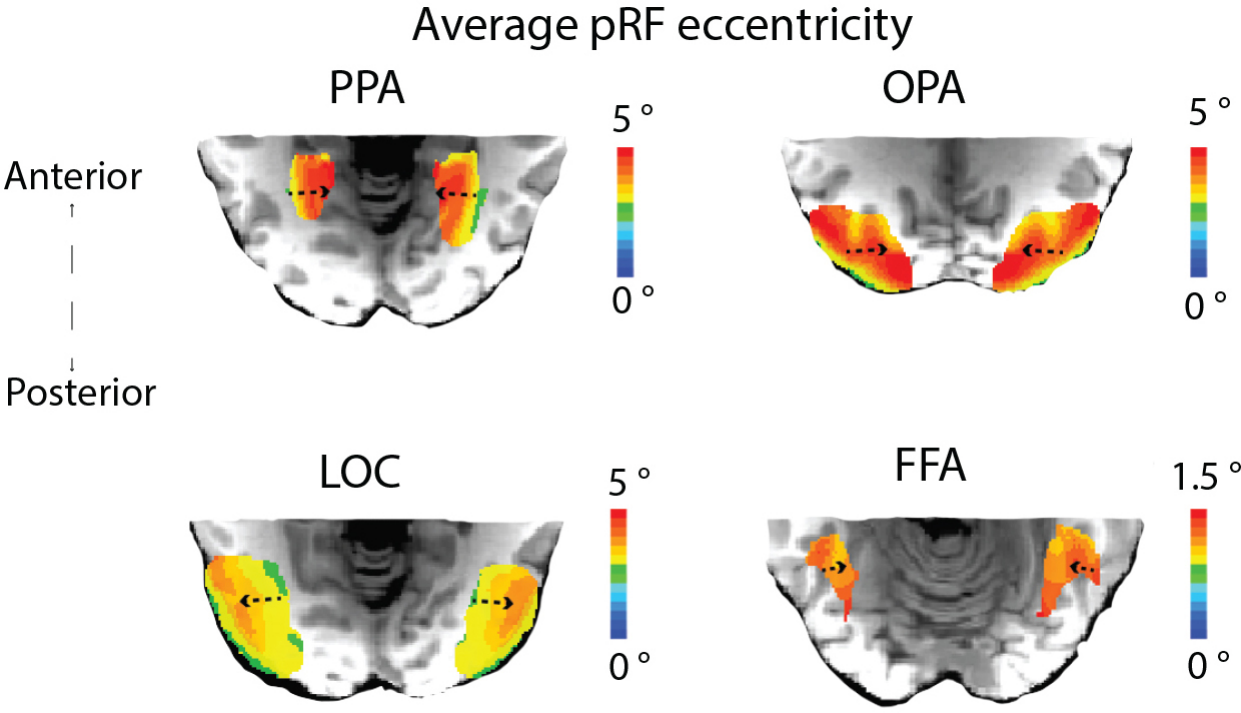
(*from left to right clockwise*): **PPA; OPA; LOC; FFA**. Organization of average eccentricity for each ROI along the axial plane. Direction relative to cardinal axis and relative magnitude of gradient of increasing pRF eccentricity is indicated by arrow. Gradient of pRF eccentricity was significant across all ROIs, *p* < .001.

**Fig. 3c.**
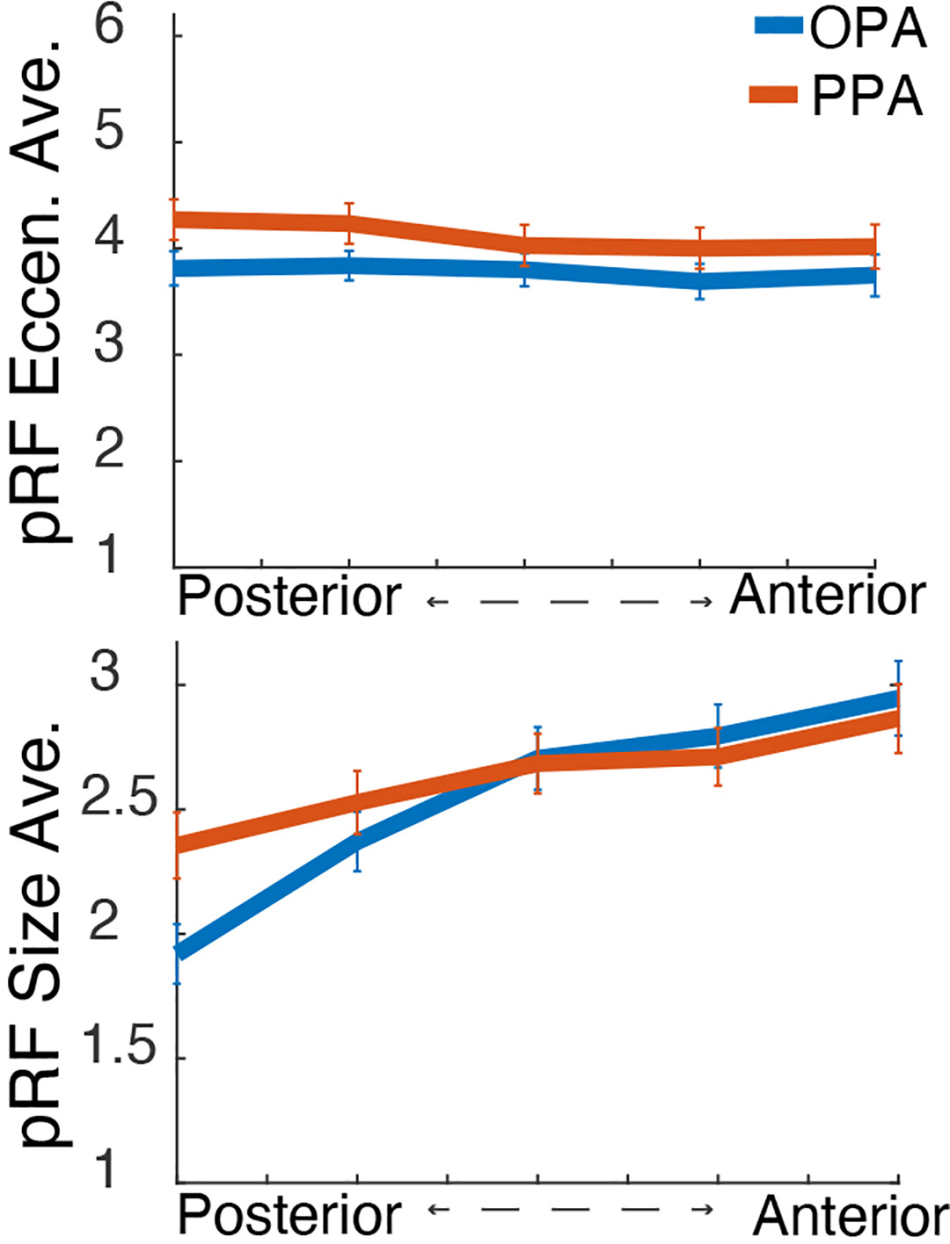
pRF eccentricity (*top*) and pRF size (*bottom*) average across HCP participants, 5 normalized posterior-anterior segments for functionally defined scene ROIs.

If we were to follow the argument that larger receptive fields translate to a stronger representation of low spatial frequencies, we would expect low spatial frequency information to be represented more in the anterior than in the posterior portions of the ROIs. In contrast, high spatial frequency information would be expected to be represented more in the posterior than in the anterior sections.

### 3.3 Neural representations of spatial frequency-filtered scenes

As our goal was to examine the relationship between SF sensitivity and pRF size in scene-selective regions, we use only the OPA and PPA ROIs going forward (excluding FFA and LOC from further analyses). Since the PPA and OPA do not respond well to simple stimuli such as gratings, we used spatial frequency-filtered images of real-world scenes to probe their spatial frequency preferences. We re-analyzed the fMRI data from Berman et al. (2017), who showed participants images of scenes from four categories (beaches, forests, cities, and highways), filtered for high (>6 cycles per degree) and low (<0.75 cycles per degree) spatial frequencies. We divided the voxels in each ROI into two halves by (i) a median split along the A-P axis, or (ii) a median split by their pRF size. The median within the PPA along the anterior-posterior axis was located within the PH-2 segment across the majority of participants relative to the probabilistically defined locations from the Wang et al. (2015) atlas.

We first analyzed the univariate BOLD activation. Secondly, we attempted to decode scene categories from high and low spatial frequency-filtered images using these two types of splits.

#### 3.3.1 Neural representations of scenes along the A-P axis

**Fig. 4.**
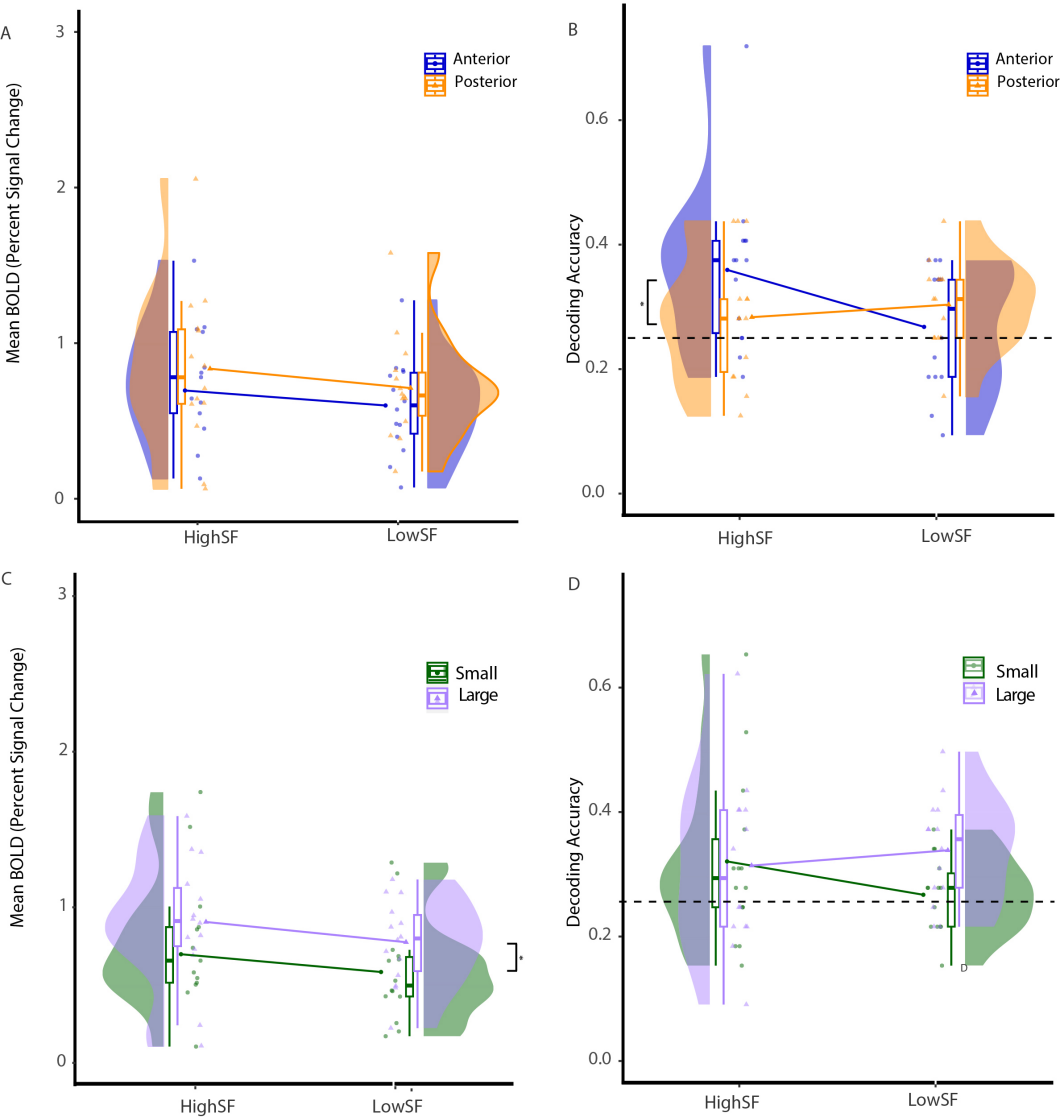
Univariate activation and decoding accuracy in PPA for low and high spatial frequency filtered scene images. The univariate activation across the anterior vs. posterior median split within PPA showed no significant main effects (A). Decoding accuracy is significantly greater for high SF when dividing voxels according to an anterior vs. posterior median split within anterior PPA (B), which suggests that the measured pRF size is not as indicative of perceptual representation differences as the anterior-posterior organizational divide. The univariate activation across the small versus large pRF showed a significant difference, where larger pRFs showed greater activation than small pRFs (C). Decoding accuracy is not significantly different for the small vs. large split of voxels in PPA (panel D), which further emphasizes the importance of the AP anatomical functional divide. Each dot indicates the average value over the voxels for an individual participant.

A 2 x 2 analysis of variance (ANOVA) of the mean BOLD signal within the PPA found no main effects for the A-P split (anterior vs. posterior: *F*(1,13) = 2.74, *ηp^2^* = 0.17, *p* = .12) or for spatial frequency (high vs. low: *F*(1,13) = 2.58, *ηp^2^* = 0.17, *p* = .13) and no two-way interaction (*F*(1,13) = 1.28, *ηp^2^* = 0.09, *p* = .28) in the PPA, Figure 4A.

For the accuracy of decoding scene categories, the ANOVA also found no main effect for anatomical subdivision (anterior vs. posterior: *F*(1,13) = 1.27, *ηp^2^* = 0.09, *p* = .28) or spatial frequency (high vs. low: *F*(1,13) = 1.701, *ηp^2^* = 0.12, *p* = .22) in the PPA. There was, however, a significant two-way interaction (*F*(1,13) = 9.018, *ηp^2^* = 0.41, *p* = .01). Tukey post hoc tests showed a significant difference in decoding accuracy between the high and low spatial frequency-filtered images within anterior PPA, where the decoding accuracy for high SF images was significantly greater than the decoding accuracy for low SF images (*M_diff_* = 0.0915, *SE* = 0.0307, *p* = .0243, CI = ± 0.099). The decoding accuracy was not above chance level for low SF images in anterior PPA, *t*(13) = 0.667, *p* = .258, but was above chance for high SF images, *t*(13) = 3.206, *p* = .0026 (see Fig. 4C). Note that this result is a replication of results reported by Berman et al. (2017).

**Fig. 5.**
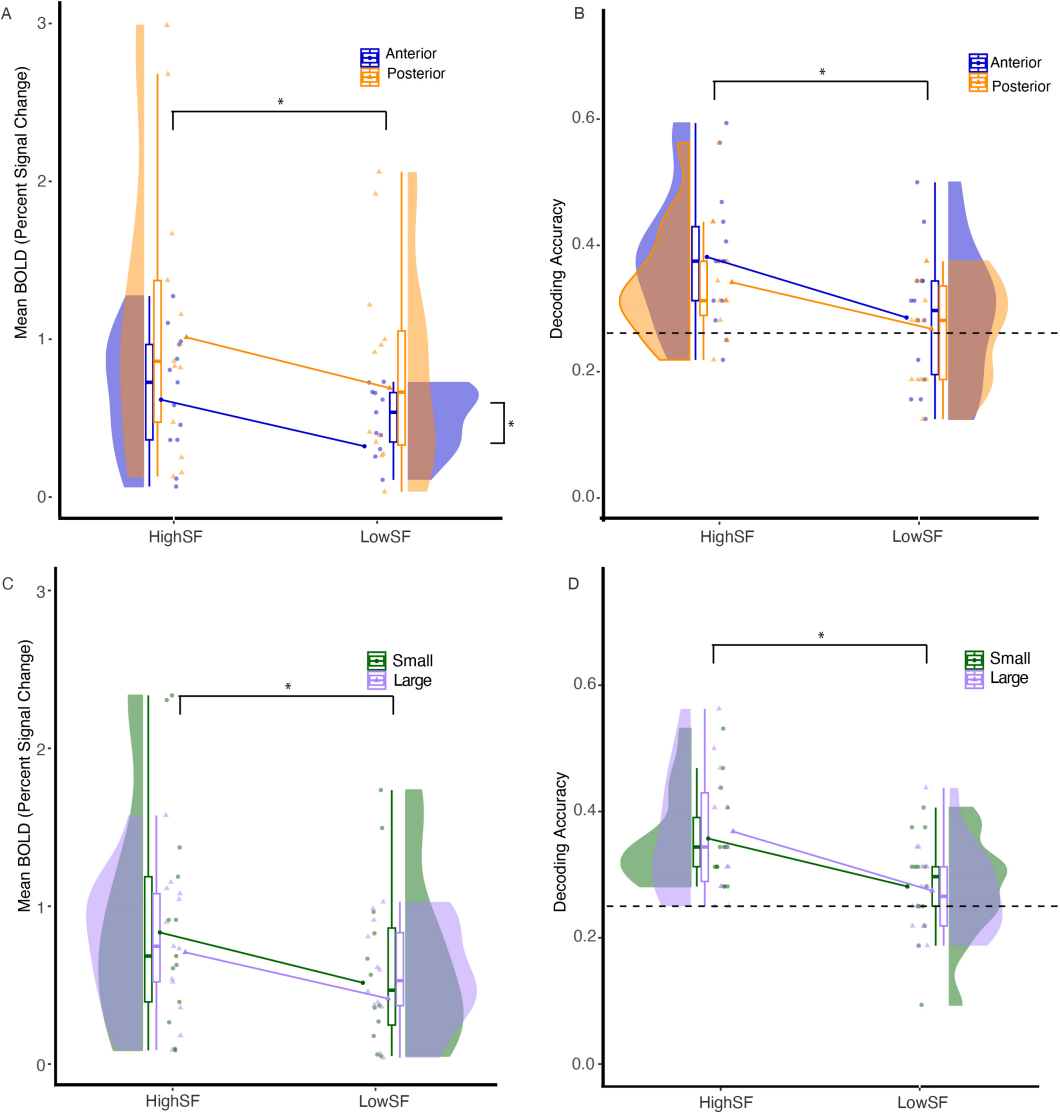
Univariate activation and decoding accuracy in OPA for low and high spatial frequency filtered scene images. Decoding accuracy and univariate activation were significantly greater for high SF when dividing voxels according to both an anterior vs. posterior median split and the pRF size within OPA (A through D). Univariate activation showed a significant main effect for anterior versus posterior (A). Univariate activation and decoding accuracy were not significant for the small versus large split of voxels in OPA (panels C and D, respectively), *p < .05.

Mean BOLD activity within OPA showed a main effect for anterior versus posterior split: *F*(1,13) = 7.573, *ηp^2^* = 0.37, *p* = .0165, with a lower BOLD signal in the anterior than the posterior OPA (*M_diff_* = 0.040). There was a main effect for spatial frequency (high vs. low: *F*(1,13) = 6.565, *ηp^2^* = 0.5, *p* = .0236), where the signal for high spatial frequency images was greater than for low spatial frequency, *M_diff_* = 0.308, Figure 5D. There was no two-way interaction (*F*(1,13) = 0.205, *ηp^2^* = 0.02, *p* = .658).

Decoding accuracy showed no significant difference for the A-P anatomical division of the OPA (*F*(1,13) = 0.978, *ηp^2^* = 0.07, *p* = .34). There was, however, a main effect for spatial frequency (*F*(1,13) = 17.11, *ηp^2^* = 0.57, *p* = .0012). Decoding accuracy was significantly higher for high than low spatial frequency-filtered images (*M_diff_* = 0.085). There was no two-way interaction (*F*(1,13) = 0.206, *ηp^2^* = 0.02, *p* = .66), Figure 5B.

T-tests showed that within anterior OPA, decoding accuracy was significantly above chance for high SF images, *t*(13) = 4.547, *p* < .001, but not for low SF images, *t*(13) = 1.228, *p* = .1207. Similarly, within posterior OPA, high SF images were decodable above chance accuracy level, *t*(13) = 3.751, *p* = .0012, but not low SF images *t*(13) = 0.834, *p* = .208.

#### 3.3.2 Neural representations of scenes by population receptive field size split

According to the view that there is a direct connection between receptive field size and spatial frequency selectivity, we would expect that voxels with large pRFs show higher decoding accuracy for low spatial frequency images and voxels with small pRFs higher decoding accuracy for high spatial frequency images. We found no indication of such a connection in the PPA or the OPA after splitting the voxels according to the median size of pRFs, which was 2.63 and 2.45 degrees of visual angle, in the right and left PPA of the group-averaged HCP data, respectively, and 2.414 and 2.215 in the OPA for the right and left hemispheres, respectively.

We performed a univariate analysis of the mean BOLD signal change. Within PPA, we found a main effect for pRF size (small vs. large: *F*(1,13) = 8.633, *ηp^2^* = 0.40, *p* = .0115), with a larger BOLD signal for large pRFs (*M_diff_* = 0.199). No main effect for spatial frequency (high vs. low: *F*(1,13) = 2.531, *ηp^2^* = 0.16, *p* = .136), and no two-way interaction (*F*(1,13) = 0.178, *ηp^2^* = 0.01, *p* = .68), Figure 4C.

Within the same voxels, an ANOVA for decoding accuracy showed no main effect for pRF size (small vs. large: *F*(1,13) = 3.535, *ηp^2^* = 0.21, *p* = .0827) or spatial frequency (high vs. low: *F*(1,13) = 0.218, *ηp^2^* = 0.02, *p* = .649) and no two-way interaction (*F*(1,13) = 3.876, *ηp^2^* = 0.23, *p* = .071) in the PPA.

Univariate BOLD activity within OPA showed no main effect for pRF size (small vs. large: *F*(1,13) = 1.08, *ηp^2^* = 0.08, *p* = .318) but it did show a main effect for spatial frequency (high vs. low: *F*(1,13) = 6.421, *ηp^2^* = 0.33, *p* = .0249). The signal for high spatial frequency images was greater than for low spatial frequency, *M_diff_* = 0.306. There was no two-way interaction (*F*(1,13) = 0.48, *ηp^2^* = 0.04, *p* = .501), Figure 5B.

For decoding accuracy, we found no main effect for pRF size (*F*(1,13) = 0.018, *ηp^2^* = 0.001, *p* = .895), in the OPA, but we did find a main effect for spatial frequency (*F*(1,13) = 14.06, *ηp^2^* = 0.52, *p* = .00243). Decoding accuracy was higher for high than low spatial frequency-filtered images (*M_diff_* = 0.0848). There was no two-way interaction in the OPA (*F*(1,13) = 0.159, *ηp^2^* = 0.01, *p* = .696).

### 3.4 Control analysis: Primary visual cortex

Since we found no indication of a connection between population receptive field size and spatial frequency preference in the PPA and the OPA, we wanted to ensure that our methods would be able to detect such a connection if it existed. We used primary visual cortex (V1) as a control region. We localized V1 using an atlas-based map (Wang et al., 2015) and projected the V1 map into individual subject space. Within V1, we divided the voxels into two groups by their population receptive field size that matched the foveola (pRF size = 0.16 degrees of visual angle) and repeated the decoding accuracy analysis as we did for PPA and OPA.

**Fig. 6.**
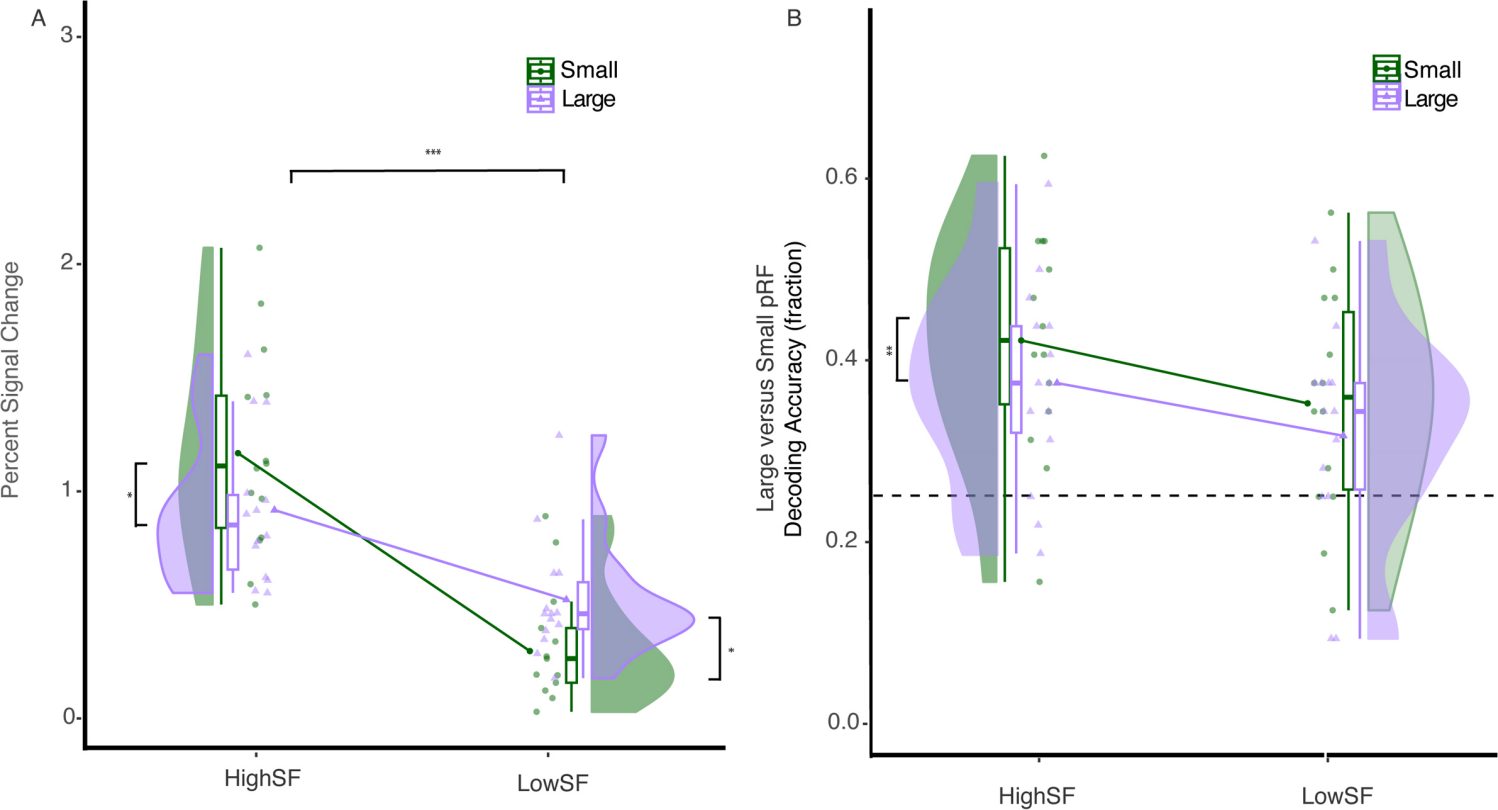
(A) Percent signal change in V1 for small and large pRF voxels; Decoding accuracy for V1, low and high spatial frequency filtered scene images decoded within small pRF voxels and within large pRF voxels (B). Univariate analysis of mean BOLD signal change in V1 showed a significant main effect for spatial frequency, and a significant two-way interaction. We found a significant main effect for pRF size with higher decoding accuracy for small voxels than large pRFs in V1, *p < .05, **p < .01, ***p < .001.

Univariate analysis of mean BOLD signal change in V1 did not yield a significant main effect for population receptive field size (*F*(1,13) = 0.016, *ηp^2^* = 0.001, *p* = .902), but it did show a significant main effect for spatial frequency (*F*(1,13) = 70.16, *ηp^2^* = 0.84, *p* < .001) and a significant two-way interaction (*F*(1,13) = 29.55, *ηp^2^* = 0.69, *p* < .001). Post-hoc Tukey tests showed significantly larger signal change for *high-*frequency images in small versus large population receptive fields, *M_diff_* = 0.251, *p* = .025, 95% Tukey’s value of CI = ± 0.318, as well as significantly larger signal change for *low-*frequency images in large versus small population receptive fields, *M_diff_* = -0.226, *p* = .042, 95% Tukey’s value of CI = ± 0.394, Figure 6A. This result confirms the commonly stated connection between receptive field size and spatial frequency selectivity in early visual cortex.

For decoding the categories of spatial frequency-filtered scene images, we found a significant main effect for population receptive field size (*F*(1,13) = 16.65, *ηp^2^* = 0.56, *p* = .001), with higher decoding accuracy for voxels with small than large population receptive fields, *M_diff_* = 0.041. There was no main effect for spatial frequency (*F*(1,13) = 2.286, *ηp^2^* = 0.14, *p* = .154) and no significant two-way interaction (*F*(1,13) = 0.432, *ηp^2^* = 0.03, *p* = .522), Figure 6B.

## 4 Discussion

We sought to test the relationship between spatial frequency representations in high-level visual cortex and pRF properties along the anterior-posterior axis in scene-selective areas. To this end, we examined the retinotopic properties by showing the anatomical organization of pRF size (Fig. 3a) across OPA and PPA. We tested the relationship between the pRF size and representations of scene content in high- versus low-spatial frequencies, with an additional control analysis in V1. We include topographic visualizations of the distributions of pRF size and eccentricity in Figure 3a and b.

Firstly, we found a significant contralateral hemifield bias across the high-level ROIs, as was expected given the contralateral organization of the visual system. Contralateral organization is characteristic of retinotopic visual areas, especially within the early visual regions of striate cortex, such as V1 and V2 (Benson et al., 2014; Dumoulin & Wandell, 2008; Sereno et al., 1995; Tootell et al., 1997). Retinotopic organization within PPA remains evident via the contralateral hemifield bias (Arcaro et al., 2009; MacEvoy & Epstein, 2007; Silson et al., 2015) and the organization of small to large pRFs going from posterior to anterior PPA, similar to what is found in lower-level visual areas. There is a clear relationship between the most densely sampled retinotopic location within the upper hemifield of PPA and the statistically greater contrast energy in the upper hemifield of scene images (Bruce & Tsotsos, 2006). These findings support the relationship between pRF organization and the natural distribution of scene image features in PPA.

Retinotopic organization is also evident via the correlation between eccentricity and receptive field size, which has been shown across visual cortex (Kay et al., 2013). These characteristics suggest that spatial frequency representations on the viewing plane would be conveyed according to pRF size within scene-selective areas, which is supported by the inverse relationship between pRF size and spatial frequency: Larger pRFs would represent low SF as the information is sampled at a lower frequency per surface area, within early visual cortex (Arcaro et al., 2009; Grill-Spector & Malach, 2004; Grill-Spector & Weiner, 2014; Hasson et al., 2002; Levy et al., 2001). However, we found no such relationship across pRF size and spatial frequency conditions in our high-level scene ROIs. In the OPA, we observed greater BOLD activity for high than low spatial frequency images, regardless of the size of a voxel’s pRF size. In the PPA, BOLD activity was significantly greater for larger pRFs across both SF conditions. Thus, the tight relationship between pRF parameters and the level of detail represented by the voxels was not found in the PPA nor in the OPA. By comparison, in our control condition, we found the expected pattern of results in primary visual cortex area V1, which showed greater BOLD activity for low SF images within voxels with larger pRFs. For high SF images, we found greater BOLD activation within voxels with smaller pRFs. Within the decoding accuracy analysis, V1 showed a main effect for the smaller pRFs voxels has significantly greater decoding accuracy than larger voxels across SF conditions.

Past research showed that contextual scene information is represented within high SF visual content during tasks (Lieber et al., 2023) and propagates from high-level visual areas to area V1 (Morgan et al., 2019; Muckli & Petro, 2013; Muckli et al., 2015; Papale et al., 2023). The significantly greater BOLD signal for high SF images in V1 is complemented by our findings of greater decoding accuracy for high SF scene images in the OPA and within anterior PPA. These results are congruent with the significantly better decoding within the smaller pRF voxels in V1. Multi-voxel pattern analysis is more sensitive to outliers and introduces nonlinearities. As such, activation patterns that are particular to a subset of voxels are less likely to disappear, as is the case when taking an average of the activation levels of BOLD signal.

Though we showed that voxels in the anterior PPA have larger receptive fields than those in posterior PPA, we found differences in decoding accuracy for the anterior-posterior split of the PPA but not the split by pRF size. This result supports the evidence that anterior and posterior PPA regions differ in their functional specialization (Baldassano et al., 2013, 2017; Berman et al., 2017): Where posterior PPA is linked more closely with processing specific scene feature properties and anterior PPA is more closely associated with processing memory-related, or mnemonic, representations of scenes (Dalton et al., 2022; Steel et al., 2021, 2023, 2024, 2025). In the OPA, by comparison, we found no difference in decoding accuracy for the splits along the AP axis or by pRF size. Our findings show that the AP axis constitutes an important organizational principle specifically for visual processing in PPA.

Representations of visual content within PPA may vary in complexity between anterior and posterior segments due to different connectivity patterns (Silson, Steel, et al., 2016). Silson, Steel, et al. (2016) observed that anterior PPA showed greater connectivity with medial place area (MPA) and the prefrontal cortex, while posterior PPA had greater connectivity with earlier areas or more posterior visual cortex, namely visual area OPA. Feedback connections from multi-sensory areas such as prefrontal cortex may transmit an abstract representation that conveys the global and contextual scene information that better distinguishes category-level differences (Bonner & Epstein, 2021). The representation of a scene in scene-selective areas seem to consist predominantly of high SF components (Walther et al., 2011; Weissman et al., 2017). Our findings of greater decoding accuracy for high SF scene images in anterior PPA where pRFs are larger, complement these previous findings, and provide further insight into the computations performed along the visual hierarchy.

The connection between receptive field size and spatial frequency that is found in V1 is not apparent within high-level scene regions. V1 neurons sample their information more directly from the retina, with only one connection in the lateral geniculate nucleus of the thalamus, which faithfully preserves retinotopic organization. Neurons with small receptive fields receive input from the foveal region of the retina, which boasts the highest density of photoreceptors. As a result, these neurons tend to be particularly sensitive to high spatial frequencies. V1 neurons at higher eccentricity receive input from more peripheral parts of the retina with lower photoreceptor density. As a result, they are better tuned to low spatial frequencies.

Unlike the early visual areas, such as V1, the PPA predominantly receives input from mid-level visual regions such as V4, as well as more abstract, higher-level regions such as prefrontal cortex (Baldassano et al., 2013). Input at higher-level cortical areas is not strictly visual, and as such the connection between receptive field size and spatial frequency preference that exists for V1 no longer holds for higher-level visual regions. Instead, other organizational characteristics, such as the AP division of the PPA, influence the feature sensitivity of the neurons.

The apparent contradiction that large pRFs can hold representations of high SF or fine-grained features is resolved if pRFs in anterior PPA are strictly connected with small, high-resolution pRFs within the early visual cortex. The hierarchical framework of visual processing, however, posits that PPA is not directly connected to the small pRFs in V1.

A limitation in the current study is that the retinotopic mapping and the spatial frequency analysis were performed in two different sets of participants. Since Berman et al. (2017) did not acquire retinotopy data for the 14 subjects in their dataset, we projected the group-level pRF parameters that we obtained from the HCP dataset to these participants. The high-resolution 7T data for 157 of the HCP participants afforded us with high-quality, high-power pRF fits at the group level. As such, while uncertainty from individual variability could affect our results, we believe that this shortcoming is mitigated by the high quality of the pRF data. Relatively few subjects (*N* = 14) were analyzed for the frequency filtered scene analysis, which could result in low-powered statistics. Though our reported effect size results indicate that the power of our analyses was generally relatively good, the null effects we reported might be due to a type II error caused by underpowered statistics.

In summary, previous research claimed that scene-selective areas such as PPA performed only coarse-grained image processing, and this was predominantly attributed to its relatively large and peripherally biased pRFs (Arcaro et al., 2009; Hasson et al., 2002). As our results show, however, large pRFs do not imply coarse global scene image processing that was expected within larger pRFs. We found that PPA had a range of smaller pRFs predominantly within the posterior PPA segment from a median split along the A-P axis (Fig. 1). Our results suggest that global scene processing does not preclude high-resolution processing within the anterior PPA. The greater decoding accuracy of high SF images within anterior PPA (Berman et al., 2017) suggests that PPA is, in fact, able to process contour features at a fine-grained scale; larger size of receptive field in anterior PPA resulted in greater decoding accuracy of high spatial frequency filtered images. These findings contradict the assumption that throughout the visual system, a given voxel’s pRF size will directly correspond with features on the viewing plane, as is the case in early visual areas, such as in area V1 (Aghajari et al., 2020; Broderick et al., 2022; Henriksson et al., 2008; Sceniak et al., 1999). Here, we showed that, while there is a correspondence between spatial frequency processing and pRF size within V1 (i.e., higher BOLD response for low SF images within voxels with larger pRFs, and for high SF images within voxels with smaller pRFs), we did not find such a relationship in the scene-selective areas PPA and OPA. Our findings suggest that retinotopic organization cannot be used to infer the resolution of the feature information being processed within high-level scene areas. Rather, populations of neurons represent information that is more complex than that which can be mapped directly from the viewing plane. Within anterior PPA, the pRF properties of the voxels exhibit less correspondence with the spatial frequency of the image on the viewing plane, compared with posterior PPA. These results present an avenue for investigating the transformation of visual features along the visual hierarchy, whether that transformation comes from top-down inputted context-dependent schemas, or primarily through early-level visual cortex as a strictly feed-forward hierarchical process.

## Supplementary Material

Supplementary Material

## Data Availability

Data are available at: https://osf.io/gwxev.
