# Supplementary material for: Population receptive field size does not correspond to spatial frequency processing in scene-selective cortex
Source: Imaging Neurosci (Camb). 2026 Jan 29;4:IMAG.a.1111. doi: 10.1162/IMAG.a.1111 (PMC12856939; doi:10.1162/IMAG.a.1111)
Supplement: Supplementary Material [file IMAG.a.1111_supp.pdf]

Supplementary Figures

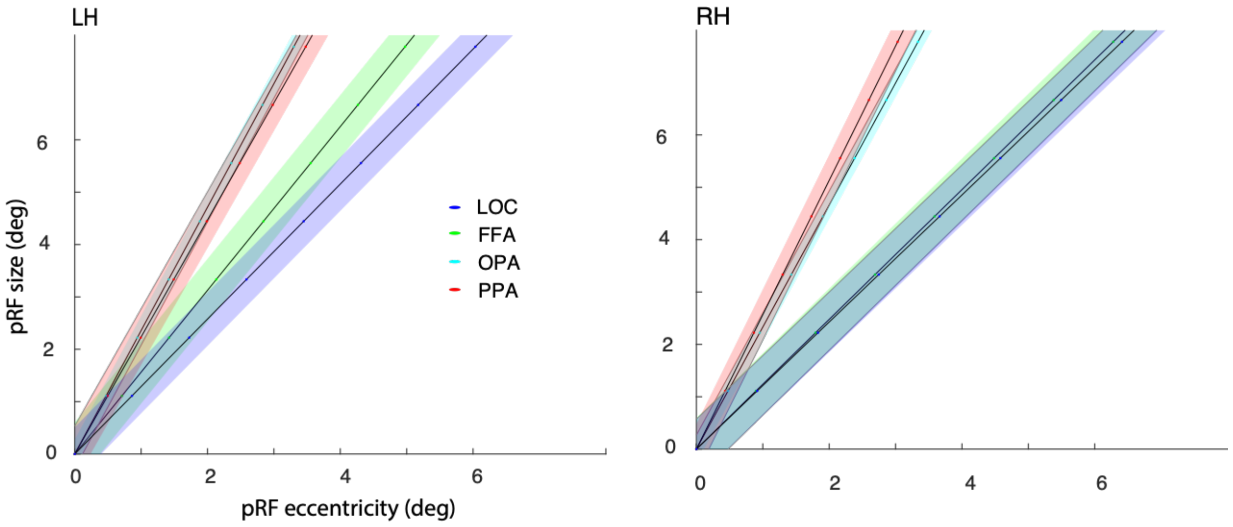

Supplementary Figure 1. Average slope of relationship between pRF size and eccentricity across HCP participants and ROIs.

Table 1.

	ROI	Axis L-M	Axis P-A	Axis I-S
Size	*PPA*	β: 0.0338, *df*: 104851, *t*: 37.69	β: 0.0466, *df*: 105818, *t*: 113.69	β: 0.0506, *df*: 106144, *t*: 63.99
	OPA	β: -0.0529, *df*: 588166, *t*:-159.8	β: 0.0438, *df*: 593278, *t*: 146.25	β: 0.0541, *df*: 593945, *t*: *223.34*
	LOC	β: -0.0642, *df*: 371662, *t*:-139.34	β: 0.0288, *df*: 373275, *t*: 74.11	β: 0.0614, *df*: 373734, *t*: 175.72
	FFA	β: 0.00956, *df*: 80223, *t*: 14.815	β: 0.01820, *df*: 82024, *t*: 48.128	β: 0.00489, *df*: 82023, *t*: 6.977
Ecc	*PPA*	β: 0.166, *df*: 105751, *t*: 105.92	β: 0.0121, *df*: 106118, *t*: 16.89	β: 0.144, *df*: 106214, *t*: 104.61
	OPA	β: 0.0600, *df*: 583253, *t*: 129.92	β: 0.0045, *df*: 592319, *t*: 10.74	β: 0.0647, *df*: 593591, *t*: 191.51
	LOC	β: -0.0732, *df*: 369387, *t*:-126.81	β: -0.0072, *df*:372616, *t*:-14.795	β: 0.0749, *df*: 373690, *t*: 170.74
	FFA	β: 0.042, *df*: 81464, *t*: 59.58	β: 0.00993, *df*: 82009, *t*: 24.066	β: -0.0064, *df*: 82014, *t*:-8.373

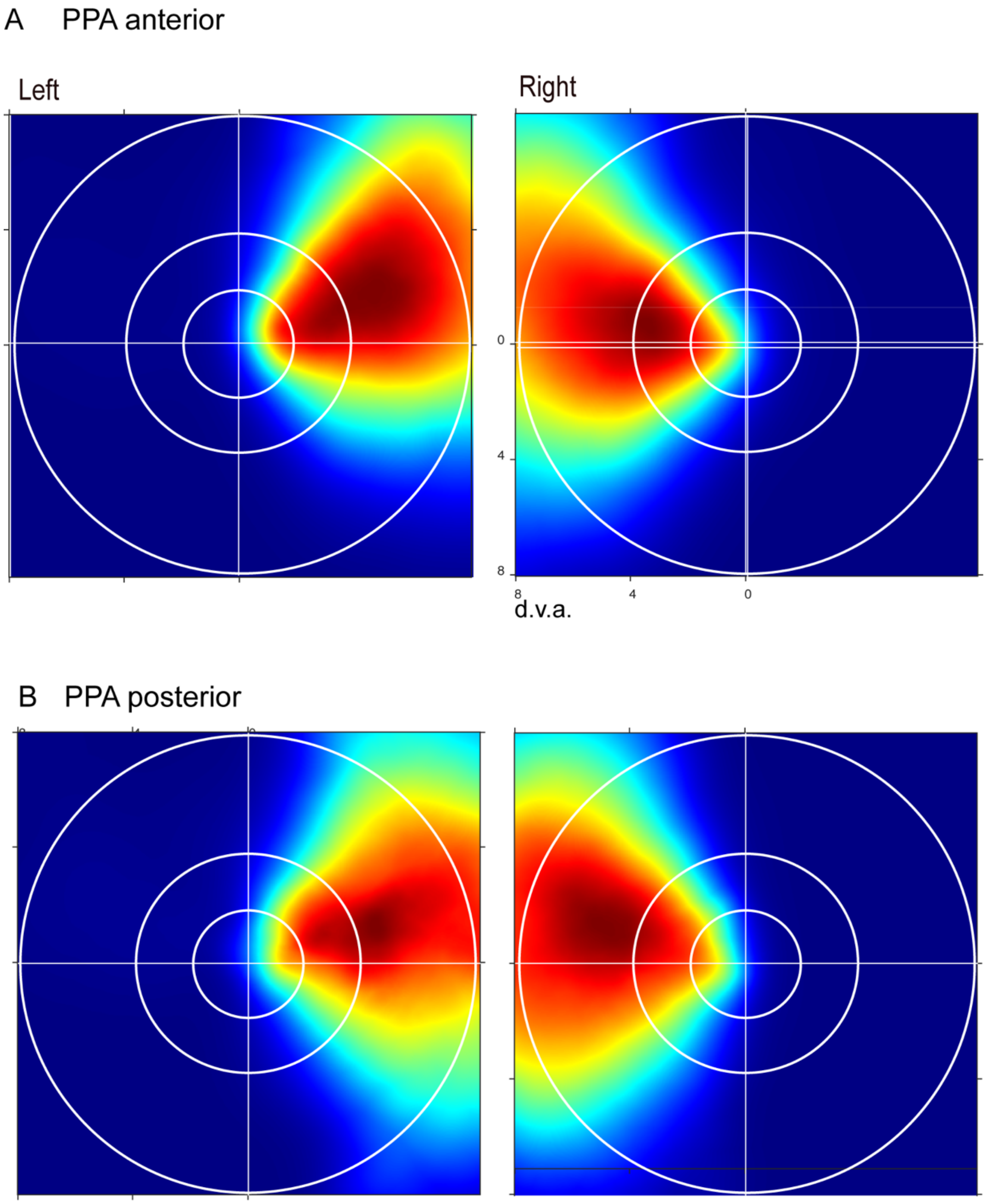

Supplementary Figure 2a (top) and 2b (bottom): PPA anterior and posterior median split division. PPA showed no difference in its upper hemifield bias between the anterior and posterior segments: p = .144, t(85) = 1.473. M_{diff} =0.049(0.311). PPA anterior segment (A) showed an upper hemifield bias: p < .001; M_{diff} = 0.512(0.405); t(86) = 11.814. PPA posterior segment (B) showed an upper hemifield bias: p < .001; M_{diff} = 0.458(0.444), t(85) = 9.558.

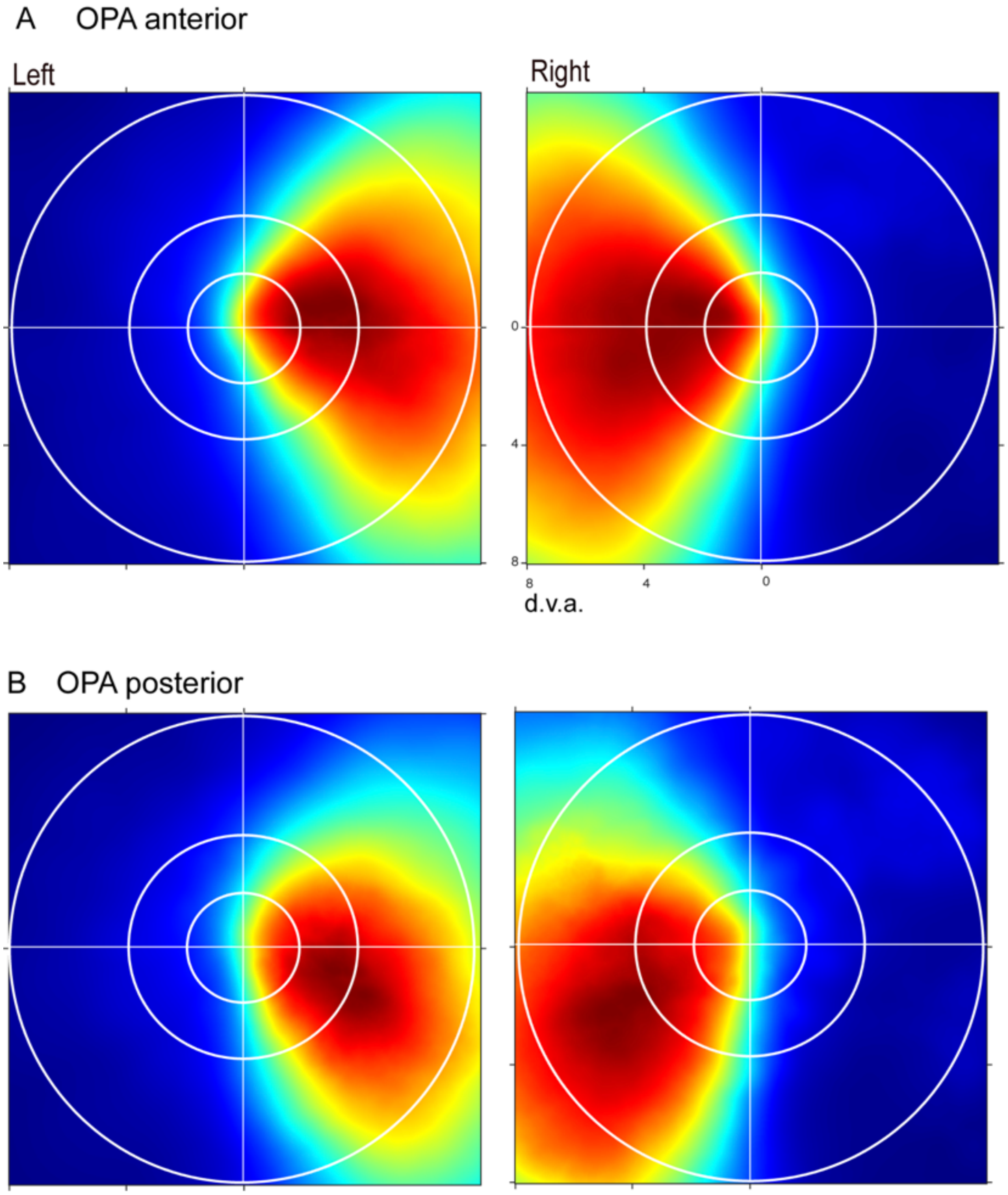

Supplementary Figure 3a (top) and b (bottom): OPA anterior and posterior median split division. We found a significantly different lower hemifield bias in posterior OPA than in the anterior segment of OPA: $p < .001$, $t(146)= 6.894$, $M_{diff} = 0.1756(0.3088)$. The anterior OPA segment (A) shows no upper or lower hemifield bias: $p = .289$; $t(146)= 1.065$, $M_{diff} =0.035(0.397)$. The posterior segment (B) shows a significant lower hemifield bias: $p < .001$, $t(146)= -4.069$, $M_{diff} = -0.141(0.419)$.
